# Pattern of Papulosquamous Disorders in Children: A Clinico-Epidemiological Study

**DOI:** 10.7759/cureus.21194

**Published:** 2022-01-13

**Authors:** Jagriti Gandhi, Surbhi Agrawal, Shreya Gupta, Kapila Verma, Anil Mohite

**Affiliations:** 1 Department of Dermatology, LN Medical College, Bhopal, IND

**Keywords:** pediatric dermatology, seborrheic dermatitis, lichen nitidus, lichen striatus, lichen planus, gianotti-crosti syndrome, pityriasis rosea, psoriasis, papulosquamous disorders

## Abstract

Introduction

Skin disorders are a major health problem in the pediatric age group and are associated with significant morbidity. Papulosquamous disorders, forming a major part of the skin diseases in children, present in a variety of clinical pattern. This study is conducted in order to study the hospital-based prevalence of papulosquamous disorders in the pediatric age group (2-14 years) and to determine the morphology and clinical patterns with respect to their age and sex distribution.

Methodology

An analytical cross-sectional study was conducted from December 1, 2019, to May 30, 2021, in the outpatient department of the Department of Dermatology, Venereology, and Leprology, JK Hospital and LN Medical College, Bhopal, India. Ninety-five consecutive patients belonging to the age group of 2-14 years, attending the Dermatology OPD and also referred cases from the Pediatrics Department were enrolled in the study. A detailed history of illness, regarding age, duration, onset, symptoms, recurrence, family history of the disease, pre-existing medical conditions, and drug intake history was taken. Information regarding the history of fever, sore throat, and vaccination was noted. Clinical and dermatological examination including hair, nail, and mucosal examination was done for all the cases. Necessary investigations were ordered for relevant cases and the data was recorded in a form specially designed for the study.

Results

In the present study, papulosquamous disorders constituted 2.9% of all pediatric (2-14 years) dermatosis. Of the various papulosquamous disorders found, psoriasis was the most common disease that was found (in 31.6%) followed by Gianotti-Crosti syndrome (18.9%), and lichen planus (18.9%). Males outnumbered females with a ratio of 1.48:1. The incidence of papulosquamous disorders was highest in 11-14 years of age in the present study.

Conclusion

Papulosquamous disorders account for a large number of the overall dermatoses, belonging to both the adult and pediatric populations. Due to significant changes in clinical presentation, geographical and environmental influences, treatment, and prognosis; the papulosquamous group of disorders in children require a varying approach than adult dermatoses. More studies are required in this field to appropriately diagnose and manage pediatric papulosquamous disorders in order to reduce the disease burden and as a key to better patient care.

## Introduction

Papulosquamous disorders are skin lesions consisting of red or purple papules or plaques with scales. These constitute a common group among the wide spectrum of skin diseases in children. In children, they may be due to genetic factors, viral or bacterial infections, or have an autoimmune etiology [1]. 

Papulosquamous disorders consist of a diverse group of cutaneous inflammatory conditions and form a large part of dermatology OPD. They include diseases such as psoriasis, parapsoriasis, pityriasis rosea, Gianotti-Crosti syndrome, pityriasis rubra pilaris, seborrheic dermatitis, and lichenoid disorders such as lichen planus, lichen nitidus, and lichen striatus [2]. 

These disorders have acute to chronic patterns, persisting from weeks to months, and sometimes up to years. The spectrum varies from inflammatory diseases like psoriasis that have a relapsing-remitting pattern to self-limiting diseases like pityriasis rosea to treatment-resistant diseases as parapsoriasis [3,4].

Why do we need to study these disorders?

Various clinico-epidemiological studies have been done globally to find out the overall prevalence of papulosquamous disorders in the adult population and a few of these disorders have also been studied individually [5-9]. However, there are very few published studies that report the clinico-epidemiological prevalence of pediatric papulosquamous disorders. The epidemiology, prevalence, clinical features, treatment options, and long-term clinical and psychological outcomes in children vary from those in adults. Most of these papulosquamous disorders have classical and distinct features and can yet appear confusing with atypical presentations in children [5]. Hence, our study is an attempt to add to the literature on papulosquamous disorders in the pediatric age group of 2-14 years.

## Materials and methods

A cross-sectional study was conducted in the Department of Dermatology, Venereology, and Leprology at JK Hospital and LN Medical College, Bhopal, India. This study was conducted to analyze the clinico-epidemiological pattern of papulosquamous disorders in the pediatric age group of 2-14 years. Informed consent was taken from their parents. Ethical clearance was taken from the concerned institutional ethics committee (REG. NO. ECR/1190/INST/MP/2019) with approval number LNMC&RC/Dean/2019/Ethics/084.

The study was conducted for a period of one and a half years, from December 1, 2019, to May 30, 2021.

Data collection method

Inclusion Criteria

Pediatric patients between two to 14 years of age with clinically diagnosed cases of papulosquamous disorders were included in the study.

Exclusion Criteria

The following were excluded: patients with skin diseases other than the ones listed in the study, children less than two years or more than 14 years of age, diagnosed cases of papulosquamous disorders currently on treatment for the disease, children whose parents did not give the consent.

Methodology

Ninety-five consecutive pediatric patients with papulosquamous disorders attending the outpatient department of the Department of Dermatology, Venereology, and Leprology as well as those referred from the pediatric department were taken in the study. A detailed history of illness, duration, onset, symptoms, recurrence, family history of the disease, pre-existing medical condition, and drug intake history was taken. Information regarding the preceding history of fever, sore throat, vaccination was noted. Clinical and dermatological examination including hair, nails, and oral and genital mucosa was done for all the cases.

On the basis of clinical and morphological presentation of the cases, diagnoses of various papulosquamous diseases were made. Necessary investigations were ordered for relevant cases and the data was recorded in a form specially designed for the study. To confirm the clinical diagnosis in doubtful cases and in those with atypical presentations, with informed consent from the patients, a biopsy sample was taken from the skin lesions and sent for histopathological examination.

Data were recorded on a proforma and was indexed in the master chart. Observations regarding various parameters included in the study were made and expressed in percentages. Graphical representation was done for all the recorded parameters. Results were analyzed using IBM SPSS Statistics for Windows, Version 28.0 (Released 2021, IBM Corp, Armonk, New York).

## Results

During the study period, a total of 3204 pediatric (2-14 years) patients attended the dermatology OPD. Of these, 95 children suffered from papulosquamous disorders making the prevalence of 2.9% of pediatric dermatosis.

Pattern of papulosquamous disorders in the pediatric age group

Of these 95 children with papulosquamous disorders, 30 (31.6%) were affected with psoriasis, 18 (18,.9%) with Gianotti-Crosti syndrome, 18 (18.9%) with lichen planus, 12 (12.6%) with lichen nitidus, 11 (11.6%) with pityriasis rosea, 3 (3.2%) with lichen striatus, and 3 (3.2%) with seborrheic dermatitis. This is depicted in Table 1.

Out of the 95 children, 56 (58.9%) were males, outnumbering females by 1.44:1. In females, the most common papulosquamous disorder was psoriasis (55.5%) followed by Gianotti-Crosti syndrome (17.9%) while in males, the most common was lichen planus (26.8%) followed Gianotti-Crosti syndrome (19.6%). This is depicted in Table 2. 

The age of the pediatric population under study was 2-14 years and the incidence of papulosquamous disorders was highest in 11-14 years of age (46.3%) followed by 6-10 years (35.7%). The least number of patients belonged to the 2-5 years age group (17.9%). This is depicted in Table 3.

**Table 1:** Clinical types of papulosquamous disorders (n=95)

Clinical types	Number of patients	Percentage
Psoriasis	30	31.6
Gianotti-Crosti syndrome	18	18.9
Lichen planus	18	18.9
Lichen nitidus	12	12.6
Pityriasis rosea	11	11.6
Lichen striatus	3	3.2
Seborrheic dermatitis	3	3.2

**Table 2:** Gender-wise distribution of papulosquamous disorders (n=95)

	Females (n=39)		Males (n=56)	
	No.	%	No.	%
Psoriasis	20	55.5	10	17.8
Gianotti-Crosti syndrome	7	17.9	11	19.6
Lichen planus	3	7.7	15	26.8
Lichen nitidus	2	5.1	10	17.8
Pityriasis rosea	3	7.7	8	14.3
Lichen striatus	2	5.1	1	1.8
Seborrheic dermatitis	2	5.1	1	1.8
Total	39	41.1	56	58.9

**Table 3:** Age-wise distribution of papulosquamous disorders (n=95)

	2-5 years (n=17)	6-10 years (n=34)	11-14 years (n=44)
Psoriasis	5 (29.4%)	9 (26.5%)	16 (36.4%)
Gianotti-Crosti syndrome	5 (29.4%)	8 (23.5%)	5 (11.4%)
Lichen planus	1 (5.9%)	5 (14.7%)	12 (27.3%)
Lichen nitidus	2 (11.8%)	6 (17.6%)	4 (9.1%)
Pityriasis rosea	1 (5.9%)	4 (11.8%)	6 (13.6%)
Lichen striatus	0	2 (5.9%)	1 (2.3%)
Seborrheic dermatitis	3 (17.6%)	0	0

Family history was negative in all the cases. Immunization as per the Indian Academy of Pediatrics (IAP) schedule was completed to date in all the children. Vaccination for COVID-19 was not taken by any of the cases. 

Of all the papulosquamous lesions, the majority were distributed over extremities (73.6%) followed by trunk (60%). Sixty children (66.6%) out of the 95 presented with scaling, while 35 (38.8%) had no scaling. Pruritus was the chief complaint in 77 children (81.1%), whereas 18 children (18.9%) did not present with any complaint.

**Figure 1:**
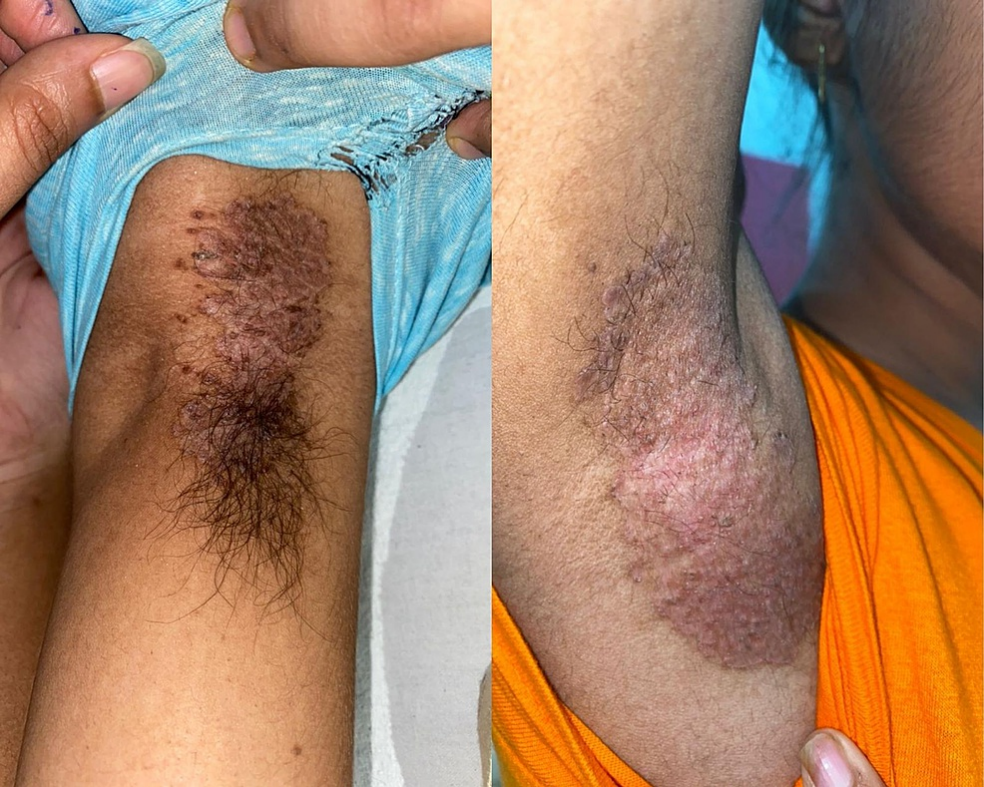
14-year-old female with inverse psoriasis plaques over bilateral axillae Single well-defined hyperpigmented scaly plaque present over left axilla before and after 20 days of treatment.

Out of these 95 pediatric cases of papulosquamous disorders, 30 were diagnosed with psoriasis, with female preponderance ( 1.5:1), the majority between 11-14 years of age. Chronic plaque psoriasis had the highest prevalence (50%), followed by scalp (20%) and guttate psoriasis (13.3%). Two out of the four patients with guttate psoriasis had a history of sore throat and one had upper respiratory tract infection. Out of the 30 psoriasis patients, 16 had nail changes, of which 13 (43.3%) had pitting and only three children (10%) had Beau’s lines. The remaining 14 cases (46.7%) had no nail changes. A cutaneous biopsy sample was taken from the scaly lesions in 11 cases to confirm the diagnosis. Major histopathological features recorded were confluent parakeratosis, rete ridge elongation and Munro’s microabscesses (11 cases), and spongiform pustules of Kogoj (seven cases). Figure 1 depicts a case of inverse psoriasis in a 14-year-old female.

Of the 95 cases, 18 had lichen planus (18.9%) with males clearly outnumbering females (3.5:1). The majority of the cases belonged in the 11-14 years age group (66.6%) followed by 6-10 years of age (27.7%). Out of all cases of lichen planus, classical lichen planus had the highest incidence (72%) followed by hypertrophic type (11.1%). Actinic lichen planus, follicular lichen planus, and eruptive lichen planus had one case (5.6%) each. Histopathology was done in nine cases and revealed vacuolar degeneration of basal layer (nine cases), pigment incontinence (six cases), band-like lymphocytic infiltrate (four cases), and wedge-shaped hypergranulosis and saw-tooth pattern ( five cases).

Twelve children had lichen nitidus (12.6%) out of the 95 cases of papulosquamous disorders, with male preponderance. The major age group affected was 10-14 years (50%) followed by 5-10 years (33.33%) . Extremities were more commonly affected than the trunk.

**Figure 2:**
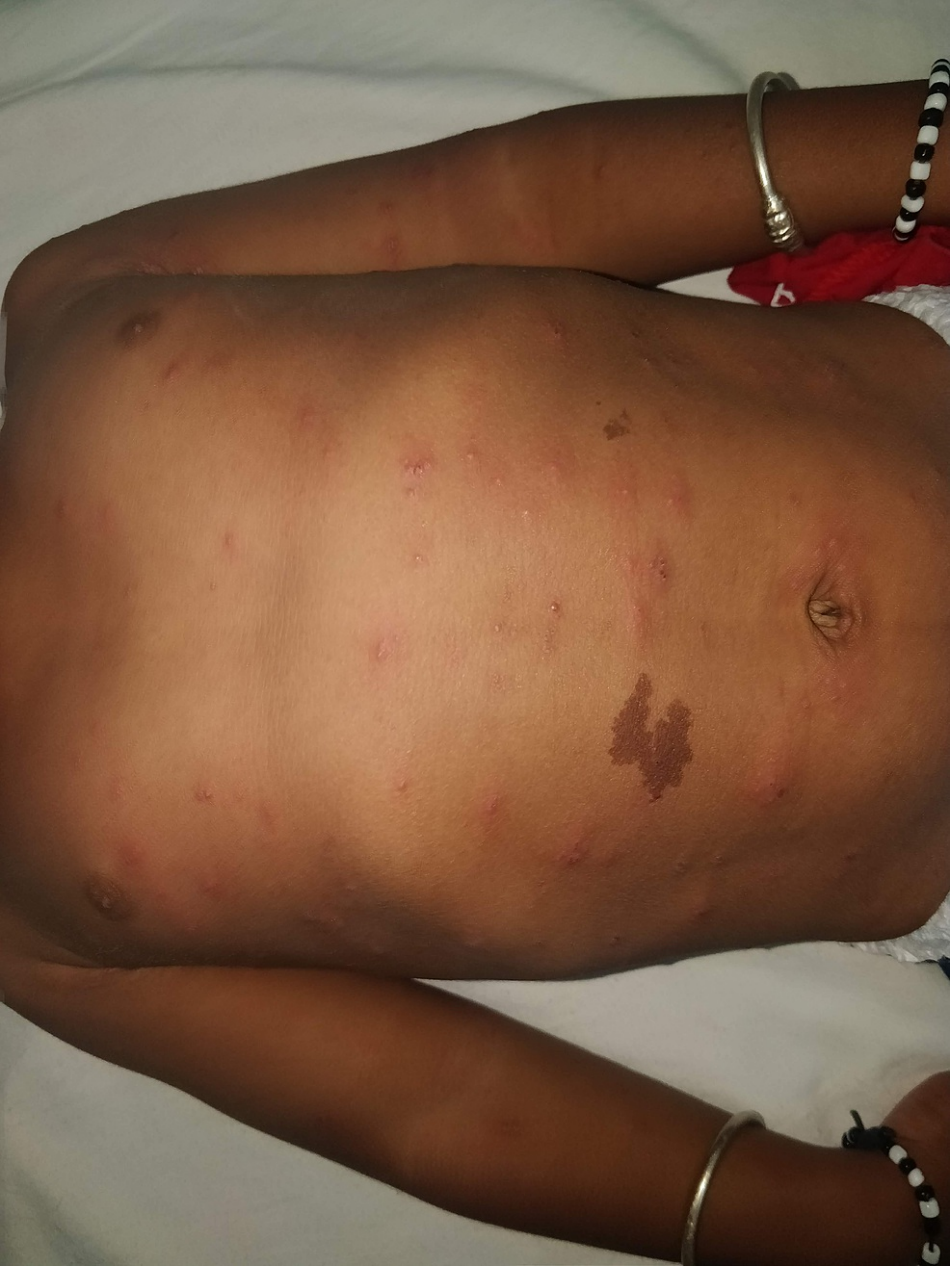
Multiple erythematous papulovesicular lesions of size 0.5*0.5cm - 0.5*1cm diameter with crusting over a few distributed symmetrically over trunk, back, bilateral upper limbs

Eighteen cases had Gianotti-Crosti syndrome with males outnumbering females (1.57:1). The majority of these belonged to the 5-10 years of age group (29.4%). The onset of eruptions in eight children (44.4%) occurred within a period of 15-20 days post-vaccination with the doses administered under the IAP immunization schedule. Five out of these belonged to the age group of 2-5 years and the rest three belonged to the age group of 5-10 years. From this, it can be surmised that vaccination could have triggered the onset of the Gianotti-Crosti syndrome eruptions. The lesions were majorly distributed over the upper trunk (Figure 2).

**Figure 3:**
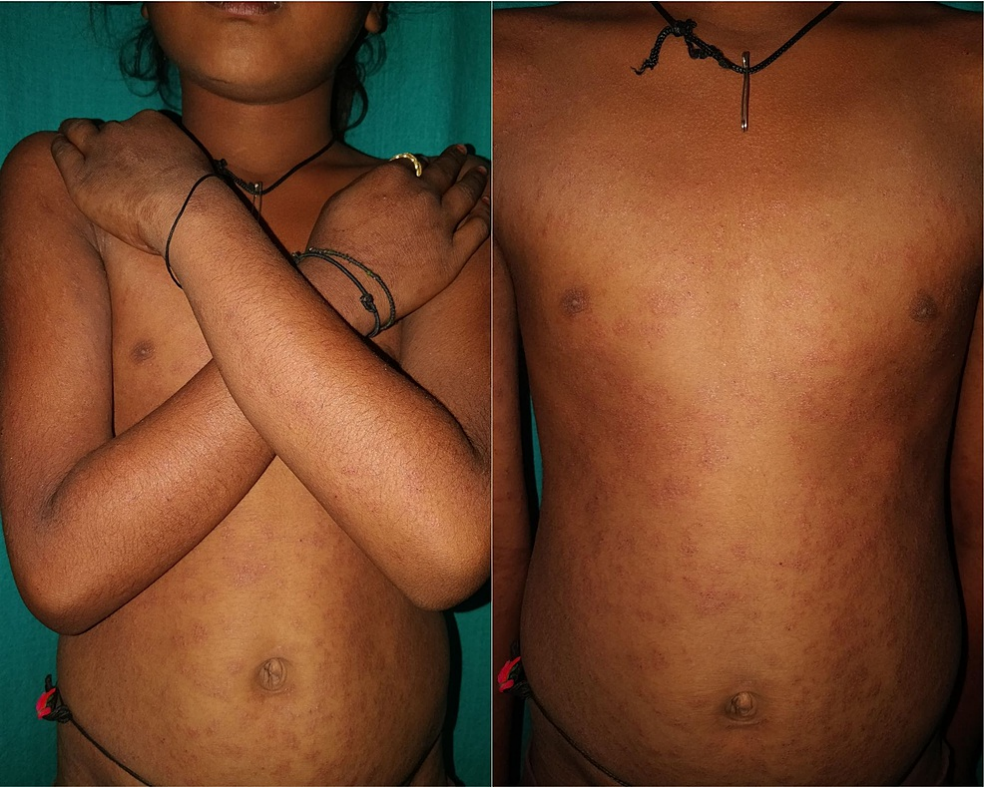
11-year-old female with multiple erythematous scaly plaques (with 'fine collarette of scales') distributed over trunk, back, bilateral upper limbs, and bilateral thighs

Eleven children (11.6%) were diagnosed with pityriasis rosea, with male predominance (72.7%). Characteristic ‘herald patch’ and ‘Christmas tree pattern’ distribution of lesions was seen in five and nine children, respectively. Figure 3 depicts a case of pityriasis rosea presenting with a typical bilateral symmetrical distribution of the scaly plaques with 'collarette of scales' over trunk and extremities. Of these 95 cases of pediatric papulosquamous disorders, three had lichen striatus and seborrheic dermatitis (3.2%) each. There were no cases of parapsoriasis and pityriasis rubra pilaris.

## Discussion

In various school-based surveys, the prevalence of skin-based diseases in various parts of India is found to range from 8.7% to 35% [10]. The prevalence of papulosquamous disorders in the present study constituted 2.9% of all pediatric dermatoses. In the study by Vetrichevvel et al. [11], the prevalence of papulosquamous disorders was 2.5% while in a study conducted in Turkey by Gul et al. [12], the prevalence was 6.9% of all pediatric dermatoses. In India, in various epidemiological studies of childhood dermatoses, the prevalence of papulosquamous disorders varied from 1.66% to 4.92% [13,14]. This is comparable to the present study. 

In comparison to other studies [14,15], the present study also included Gianotti-Crosti syndrome. Of the various papulosquamous disorders found in children, psoriasis (31.6%) had the highest prevalence followed by Gianotti-Crosti syndrome (18.9%) and lichen planus (18.9%).

In the present study, males (58.9%) outnumbered females (41.05%) with a ratio of 1.48:1. This was comparable with the study by Vetrichevvel et al. [13], where males (57.5%) predominated females. However, in the study by Vadite [15], cases of papulosquamous disorders were more in females (54.9%), which was in contrast to that seen in the present study. The incidence of papulosquamous disorders was highest in the age group of 11-15 years (46.3%) in the present study. Also, in the present study, out of the 95 pediatric cases, 60 cases presented with scaling (66.6%), and pruritus was the major complaint in 81.1% of children. This was comparable to the studies by Nyfors and Lemholt [16] and Nanda et al. [17].

In the present hospital-based study, the prevalence of childhood psoriasis was 0.93% among pediatric dermatoses. This is comparatively high compared to the study conducted in Karnataka, India [15], which had found the prevalence of psoriasis to be 0.38%. A study conducted in the South Indian pediatric population [18] has shown comparable prevalence to that of the present study, i.e. 1.4%. Of the 95 pediatric patients with papulosquamous disorders, 30 patients (31.1%) had psoriasis with females outnumbering males (2:1). These results are almost consistent with the studies by Nyfors and Lemholt [16], Morris et al. [19], and Wu et al. [20]. However, a study conducted by Kumar et al. [21] reported a slight male preponderance. Maximum psoriasis cases in the study belonged to the 11-14 years of age group in the present study, which was similar to the study by Kumar et al [21]. The most common presentation in children is chronic plaque-type, same as in adults [4]. The next most common presentation in children is guttate type psoriasis, having different trends to adults. The most common type of psoriasis in our study was chronic plaque-type (50%) followed by scalp psoriasis (20%) and guttate psoriasis (13.3%), which is comparable to study conducted by Morris et al. [19], and Wu et al. [20]. However, the study by Nyfors and Lemholt had the highest prevalence of guttate psoriasis followed by plaque-type psoriasis [16] and infection was the most commonly reported precipitating factor. However, genetic and environmental variables may play a role, as children in India are more likely to develop the disease's persistent plaque form rather than the transient guttate type. There was no family history seen in our study, in contrast to the study by Kumar et al. [21] who found positive family history in 4.5% of their patients. In our study, nail involvement was reported in 53.3% of our patients, and pitting was the most common finding (43.3%), which shows comparable findings to studies from Kuwait [22] and by Kumar et al. [21]. 

The prevalence of childhood lichen planus among the pediatric dermatoses cases in our study was 0.56%. Prevalence is low when compared to the study conducted by Handa and Sahoo in North India [23], who reported 2.5% lichen planus cases among the total pediatric patients, and another by Vadite [15] who reported a much higher frequency of 28.43%. Childhood lichen planus in the present study was 18.9%, which is comparable to the study by Vetrichevvel et al. [11] where lichen planus affected 14.8% of the children having papulosquamous disorders. In the present study, males outnumbered females as seen in studies of Handa and Sahoo [23] and Valdite [15]. Kumar et al. [21], however, reported more incidence of lichen planus in females as compared to males, which is in contrast to the present study. The most common clinical type of lichen planus in our patients was the classical form affecting 72.2% of the subjects, similar to the earlier mentioned studies [21,23,24]. In most of the studies [23-25], there was no familial history seen with lichen planus. This is true for our study as well.

In the present study, the prevalence of lichen nitidus is 12.6% of the pediatric papulosquamous cases, with a male preponderance (5:1). This is comparable with studies conducted by Zapata et al. [26] and Lapins et al [27]. Patients in the age group of 10-14 years were mainly affected. Lichen nitidus may sometimes be associated with other lichenoid dermatoses such as lichen planus and both conditions may coexist in the same patient [25]. However, there was no association seen in our study. Involvement of nail and mucosal surfaces was not observed in any of the cases.

Lichen striatus affected 3.2% of the children suffering from papulosquamous disorders, of which females predominated males (1.5:1). This was similar to the study by Vadite [15]. In a study to determine the pattern of dermatoses in children in South India, pityriasis rosea constituted 0.2% [18], whereas, in our study, the incidence was slightly higher, i.e. 0.3%. The present study reported an incidence of pityriasis rosea in 11.6% of the pediatric cases with papulosquamous disorders. Vetrichevvel et al. [11] reported pityriasis rosea as the most common papulosquamous disorder in their study, constituting 32.4% of the papulosquamous disorders presented.

Wananukal et al. reported a higher incidence of seborrheic dermatitis in males [28]. In contrast, in our study, girls (55%) outnumbered boys. This was comparable to the study by Vadite [15], in which the male to female ratio was 1:2. In our study, all of the children with seborrheic dermatitis were in the age group of 2-3 years.

Gianotti-Crosti syndrome is characterized by the presence of symmetric erythematous papular and papulovesicular eruptions over the face, extremities, and buttocks, and is commonly seen in the pediatric age group [1]. It has been classically associated with viral etiology, such as hepatitis B virus, as well as rarely with Epstein-Barr virus and cytomegalovirus [29-30]. Apart from viral infections, Gianotti-Crosti syndrome is also associated with vaccinations [30]. It formed 18.9% of the total pediatric cases with papulosquamous disorders. Males outnumbered females with a ratio of 1.57:1.0. Gianotti-Crosti syndrome commonly affects children between two to six years of age [30]. In the present study too, the majority of the cases belonged to the age group of 2-5 years (29.4%). In the present study, the onset of eruptions seen in eight children (44.4%) occurred within a period of 15-20 days post vaccination; five children were between two to five years of age and the remaining three belonged to the age group of 5-10 years. The findings of the present study lead to the surmise that vaccination could have triggered the Gianotti-Crosti syndrome. The high incidence in the 2-5 years age group might be due to more vaccination administration during this age group. Numerous findings of Gianotti-Crosti syndrome occurring in relation to vaccination have been documented in Table 4.

**Table 4:** Summary of various studies where vaccination has been related to GCS DPT: Diphtheria Pertussis Tetanus;  OPV: Oral Poliovirus Vaccines;  MMR: Measles, Mumps, Rubella; GCS: Gianotti-Crosti syndrome

Authors	Age	Country	Year	Vaccine	Virus associated
Babu and Arivazhahan [30]	18 months	India	2013	DPT and OPV	None
Kolivras and André [31]	18 months	Belgium	2008	Hepatitis A	None
Kang and Oh [32]	3 years	Korea	2003	Japanese encephalitis	None
Andiran et al. [33]	11 months	Turkey	2002	Measles and Hepatitis B	None
Velangi and Tidman [34]	15 months	United Kingdom	1998	MMR	None
Lacour et al. [35]	5 years	Switzerland	1995	MMR	Epstein-Barr virus
Baldari et al. [36]	12-15 months (five cases)	Italy	1994	DPT and OPV	Epstein-Barr virus
Retrouvey et al. [37]	19 months	United States of America	2012	DPT and Varicella	None

No cases of parapsoriasis and pityriasis rubra pilaris were reported in the present study. The low number of patients may be accountable for this.

Limitations of the study

Short study duration and small sample size were the limitations of the study. Also, environmental factors influencing various diseases have not been studied.

## Conclusions

The present study records the clinical patterns of various papulosquamous disorders in the pediatric age group mentioning prevalence, gender and age distribution, clinical subtypes and features of individual diseases, and their associations (if any). Psoriasis was the most common papulosquamous disorder reported followed by lichen planus and Gianotti-Crosti syndrome.

Papulosquamous disorders account for a large number of the overall dermatoses, belonging to both the adult and pediatric populations. However, there is a dearth in the studies reporting the clinical patterns of this group of disorders in pediatric population. Therefore, more studies are required in this field to appropriately diagnose and manage the pediatric papulosquamous disorders in order to reduce the disease burden and as a key to better patient care.
